# Gauging Working Memory Capacity From Differential Resting Brain Oscillations in Older Individuals With A Wearable Device

**DOI:** 10.3389/fnagi.2021.625006

**Published:** 2021-02-19

**Authors:** Soheil Borhani, Xiaopeng Zhao, Margaret R. Kelly, Karah E. Gottschalk, Fengpei Yuan, Gregory A. Jicha, Yang Jiang

**Affiliations:** ^1^Department of Mechanical, Aerospace, and Biomedical Engineering, University of Tennessee, Knoxville, Knoxville, TN, United States; ^2^Sanders-Brown Center on Aging, College of Medicine, University of Kentucky, Lexington, KY, United States; ^3^Center on Gerontology, School of Public Health, University of Kentucky, Lexington, KY, United States; ^4^Department of Audiology, Nova Southeastern University, Florida, FL, United States; ^5^Department of Neurology, College of Medicine, University of Kentucky, Lexington, KY, United States; ^6^Department of Behavioral Sciences, College of Medicine, University of Kentucky, Lexington, KY, United States

**Keywords:** working memory, EEG, resting-state, mild cognitive impairment, coherence analysis

## Abstract

Working memory is a core cognitive function and its deficits is one of the most common cognitive impairments. Reduced working memory capacity manifests as reduced accuracy in memory recall and prolonged speed of memory retrieval in older adults. Currently, the relationship between healthy older individuals’ age-related changes in resting brain oscillations and their working memory capacity is not clear. Eyes-closed resting electroencephalogram (rEEG) is gaining momentum as a potential neuromarker of mild cognitive impairments. Wearable and wireless EEG headset measuring key electrophysiological brain signals during rest and a working memory task was utilized. This research’s central hypothesis is that rEEG (e.g., eyes closed for 90 s) frequency and network features are surrogate markers for working memory capacity in healthy older adults. Forty-three older adults’ memory performance (accuracy and reaction times), brain oscillations during rest, and inter-channel magnitude-squared coherence during rest were analyzed. We report that individuals with a lower memory retrieval accuracy showed significantly increased alpha and beta oscillations over the right parietal site. Yet, faster working memory retrieval was significantly correlated with increased delta and theta band powers over the left parietal sites. In addition, significantly increased coherence between the left parietal site and the right frontal area is correlated with the faster speed in memory retrieval. The frontal and parietal dynamics of resting EEG is associated with the “*accuracy and speed trade-off*” during working memory in healthy older adults. Our results suggest that rEEG brain oscillations at local and distant neural circuits are surrogates of working memory retrieval’s accuracy and processing speed. Our current findings further indicate that rEEG frequency and coherence features recorded by wearable headsets and a brief resting and task protocol are potential biomarkers for working memory capacity. Additionally, wearable headsets are useful for fast screening of cognitive impairment risk.

## Introduction

Visual working memory plays pivotal roles in many daily goal-directed activities, such as searching for a car in a parking lot or driving. For an individual to find the right car, one must keep task-relevant information (e.g., the color of the car) in mind as a memory target, while rejecting non-target information. The task holds true while driving, where one needs to survey the mirror for the surroundings while the car is in motion. Retrieval accuracy and speed are essential memory performance measures that reflect working memory capacity as temporary storage of information and later manipulation (Hollingworth and Beck, 2016). There are age-related changes that occur in working memory, such as slowed neural processing speed or reaction times (Wang et al., 2011).

Recent advances in machine learning algorithms and wireless technology have allowed for wearable EEGs to gain renewed traction as a means to measure brain activity (Jiang et al., 2017; Abiri et al., 2019b). EEG signals can provide information about the oscillatory activity and brain functional connectivity across long-range brain networks (Abiri et al., 2019b). Study of resting-state and task-induced non-random patterns of intrinsic brain activities brings about major advantages such as being non-invasive, lower-cost, and portable (Noh et al., 2018; Guran et al., 2019; Mapelli and Özkurt, 2019; Nyhus et al., 2019; Román-López et al., 2019). For instance, analyzing event-related potentials (ERPs), which is the averaged EEG brain response onset to a psychological event (e.g., attentional vigilance or memory retrieval), offers a brain imaging technique to gauge cognitive processes (McBride et al., 2012; Li et al., 2017; Abiri et al., 2019a; Borhani et al., 2019; Jiang et al., 2021)1). Additionally wearable EEGs allows for the recording brainwaves during rest without additional instruments to induce a series of stimuli, i.e., similar to ERP-like experiments, which may induce less fatigue and anxiety in participants. Although there are plenty of advantages there are also disadvantages to wearable EEGs. One such disadvantage is that wearable EEGs are more susceptible to excessive body movement that can cause channel noise and artifacts in the recorded EEG signals, which can negatively impact the quality of the task-induced signals.

A variety of studies have examined the characteristics of resting-state measures to explain the neurophysiology of various diseases, such as migraines and Alzheimer’s disease (AD), which further highlights the utility and quality of information obtained from rEEG measurements (Cao et al., 2016; Cassani et al., 2018). The dominant brain oscillation in awake resting-state is a low alpha oscillation (8–10 Hz), which is related to global attention. An overall increase in the low alpha power during the eyes-closed compared to the eyes-opened resting would be easily depictable. The increase is mostly manifested over parietal and occipital areas (Barry and De Blasio, 2017). Increased alpha positively correlates with higher accuracy and faster reaction times during verbal recognition tasks (Zunini et al., 2013). Resting eyes-closed EEG rhythms (e.g., posterior alpha and delta) change in pathological aging as a function of the global cognitive level (Babiloni et al., 2006). The higher alpha (10–13 Hz) is mostly correlated with sensorimotor and semantic processing related brain activities (Babiloni et al., 2010). Jeong (2004) studied brain oscillations during rest among normal aging individuals and individuals with dementia. They discovered increased delta (1–4 Hz) and theta (4–8 Hz) and decreased beta power among individuals with dementia when compared to the normal aging group. In regard to theta, Rossini et al. (2007) revealed that individuals with cerebrovascular dementia may have an overall higher theta oscillation when compared to individuals with AD.

In addition to diseased states, rEEG has been linked to cognitive performance in healthy normal adults. Analyzing resting-state EEG (rEEG) during eyes-open, a significant positive correlation between delta power captured over the left frontal and temporal regions and reaction times were observed among healthy adult participants. Additionally, higher frontal and parietal alpha correspond to lower accuracy and higher inter-trial variability of reaction times (Torkamani-Azar et al., 2020). Individuals with a lower working memory capacity have also shown larger changes between delta/theta ratios in rEEG with eyes open and eyes closed. The changes were also identified in the right posterior frontal and parietal cortices (Heister et al., 2013). Increased beta and gamma power over the right temporal and parietal areas during eyes-closed rEEG were positively correlated with second language acquisition (Prat et al., 2016), which is an indicator of working memory capacity. EEG signals’ high temporal resolution allows the evaluation of functional connectivity by estimating coupling between different pairs of independent signal sources or different electrodes across frequency bands. The connectivity can capture relationships between different brain areas, providing valuable information to discover novel neuromarkers. Magnitude-squared coherence is a normalized measure of co-activation and temporal synchronization in the spectral domain between pairs of sources, or electrodes, representing functional coupling. The measure calculated by fast Fourier Transform, illustrating how information is processed during motor and cognitive processing between the active regions.

There is a network of brain regions subserving working memory functions. Older adults with cognitive impairment and preclinical AD pathologies show network connectivity changes (blood-oxygen level dependent signals at different brain regions) subserving working memory functions (Jiang et al., 2016). Thus, coherence during resting-state is a potential neuromarker of different neurodegenerative diseases. Babiloni et al. (2009) investigated the coherence network during resting between normal aging, MCI, and AD cohorts. The study revealed that AD cohorts have higher coherence over delta band and lower coherence over alpha oscillations compared to MCI and normal peers. Using magnetoencephalography (MEG) brain imaging, Stam and Van Dijk (2002) indicated a decrease in total brain synchronization in the beta and gamma bands among AD cohorts compared to normal aging cohorts. Bosboom et al. (2009) demonstrated that dementia in individuals with Parkinson’s disease is positively correlated with a decrease of alpha frontotemporal coherence and a drop of local gamma oscillation during rest. Decrease of frontoparietal and interhemispheric coherence in the delta and alpha bands during rest in individuals with AD has also been depicted (Sankari et al., 2011). Overall, a decrease in the measure of coherence in individuals with AD is often associated with damage in the cholinergic system and its interactions with the intrinsic neurotransmitters’ excitation and inhibitions along pathways that connecting the frontal area to other brain regions, including temporal, occipital, and parietal areas (Scarr et al., 2013). The increase of coherence between the temporal region and other brain regions is considered a compensatory mechanism to tackle a decline in the coherence between other regions (Demirtaş et al., 2017).

Using a wireless headset with a 15 min resting and task protocol, we test the hypothesis that individuals with a higher working memory capacity show significant correlations with specific brain oscillations at local and distant neural circuits. Specifically, we explore relations between mean performance (i.e., reaction times and accuracy) of the Bluegrass memory task and the various rhythms during eyes-closed rEEG. Additionally, the connectivity between pairs of EEG electrodes in all oscillation bands were explored using magnitude-squared coherence between two EEG sites. The neural correlates of rEEG that correspond to task-dependent performance scores were measured in older adults with normal cognition. This research aimed to examine easy-to-use EEG markers to identify preclinical risk for cognitive impairments. The identification of these correlates is the first step toward early detection of preclinical changes in the brain that affects working memory (core cognitive functions). Early identification may allow for early intervention and prolongation of the maximal cognitive functioning in rapid-growing older populations.

## Materials and Methods

### Human Participants

**FIGURE 1:**
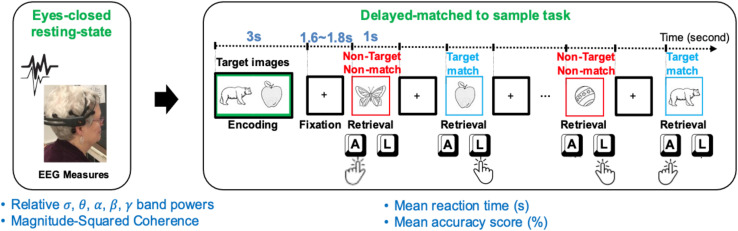
A schematic of the resting EEG (rEEG) and Bluegrass memory protocol in a right-hand dominant human participant. The participants’ eyes-opened resting-state followed by an eyes-closed resting-state followed by a memory task (a modified, delayed match-to-sample paradigm). The task includes encoding two target images in mind, then responding with the right index to indicate the memory target match (pressing “L” key on the keyboard) and left index finger for non-target in a 5-min task. During the second 5-min testing block, the participant was asked to indicate a memory target match by using the left index finger (pressing “A” key on the keyboard).

Using identical Bluegrass memory protocol, 43 community-dwelling older adults (age ≥ 60 years) including 20 males [median = 68 years, interquartile range (IQR) = 5.5], and 23 females (median = 72 years, IQR = 10) were recruited. All participants were tested at either the University of Kentucky (United Kingdom) Alzheimer’s Disease (AD) Center (United Kingdom-ADC) or Aging, Brain, and Cognition Laboratory, at the Department of Behavioral Science, College of Medicine. The average age of participants was 71.6 ± 7.0 (min: 60, max: 91). Participants had a normal or corrected-to-normal vision and were not under the influence of any drowsiness-inducing or cognitive enhancing medication. Participants were all native English speakers, and mostly right-handed (6 were left-handed). To balance dominate hand bias (faster reaction times) within each participant, the index finger of the dominated hand was used to indicate memory target in the first 5 min of the task (Figure 1). Then the person’s non-dominate hand was used to indicate memory target in the second 5-min of the task.

The experimental protocols were approved by the Institutional Review Board (IRB) of the University of Kentucky, Lexington, KY, United States. All participants provided signed informed consent in accordance with United Kingdom IRB. In other words, the recordings were carried out under the Code of Ethics of the World Medical Association (Declaration of Helsinki) for experiments involving humans.

### Wireless EEG Signal Acquisition and the Experimental Procedure

Neural data collections were performed in a quiet and dimly lit room. A water-hydrated, portable, and wireless EEG headset, Emotiv EPOC+, was used for recording all EEG signals. The headset has 14 channels over AF3, F7, F3, FC5, T7, P7, O1, O2, P8, T8, FC6, F4, F8, and AF4 according to 10–20 International Electrode Placement System, and collects brain electrical activities with a sampling rate of 128 Hz with 14 bits resolution. A low impedance (<10 KΩ) was maintained for all EEG electrodes during the experiments. EEG signals were electrically referenced using CMS/DRL references at the left/right mastoid (P3/P4). Participants were seated comfortably on a chair 50 cm from a 24-inch LCD monitor. Every participant completed a 60-s resting session with eyes open, followed by a 60-s resting session with eyes closed.

The experimental interface provided the participants with instruction and primed them with a count-down to initiate resting sessions. During the eyes opened condition, the participants were requested to stay relaxed and look straight at a fixed-in-time and pleasant scenery of the blue ocean and avoid mind wandering. During the eyes closed condition, participants were requested to stay relaxed and avoid falling asleep once resting-state sessions were completed, participants were instructed to prepare for a memory task. A delayed-match-to-sample (DMS) task was adopted to assess simultaneous visual matching ability and short-term visual recognition memory. This short-term memory task, which is a variant of the Sternberg memory-scanning task (Sternberg, 1969) has been well-studied and can modulate various cognitive processes including encoding, decision making, visuomotor selection, rehearsal, and retrieval. In this current version of the task, participants were instructed to memorize two sample images in 5 s. Two target images with a green border were initially presented for 3 s (initial memory encoding). After a jittered delay (1.6∼1.8 s), a sequence of 12 test images, including target and distractor images, were serially presented.

The number of target and distractor images were fairly distributed in each trial. Each trial lasted approximately 40 s, which included a 3-s encoding and 1-s presentation of each test image, and a fixation jittered inter-trial of 1.6∼1.8 s in between (see Figure 1). Stimuli included two-dimensional black and white pictures of familiar objects taken from Snodgrass and Vanderwart (1980). Each picture was presented with a black background in an area of 8.3 cm × 5.8 cm. All stimuli were presented on a high-resolution color monitor with a 60 Hz refresh rate. Stimuli were presented at a 65-cm visual distance and a visual angle of approximately seven degrees. Test images were normalized across retrieval status (i.e., target matching or non-matching) for image familiarity and image complexity.

All participants practiced two sample trials prior to starting the memory task. The task was performed in two blocks of eight trials each. After presenting each picture, participants were asked to respond whether the image matched one of the test images by pressing either the “A” or “L” key. During the first block, the participants responded to the match target images based on their dominant hand. For example, a right-handed individual would press the “L” key for a match and the “A” key for a non-match. The keys would be reversed for a left-handed individual- the match key would be the “A” key, and the non-match would be the “L” key. Once the first block was completed, participants were reinstructed for the second block. The second block required the match response to be from the non-dominant hand. Overall, the Bluegrass resting and memory protocol lasted approximately 15 min.

### Newly Developed Experimental Software for Sharing

To eliminate the need for commercial software, a free PsychoPy3 builder and coder software (Brooks, 2019; Peirce et al., 2019) were utilized in the design and to present visual stimuli in a timely synchronous manner. The software suite is a version of the Psychopy python library used to develop, measure, and deliver different behavioral experiments. Behavioral responses in terms of key presses along with the continuous EEG signals were recorded during the experiment. Lab Streaming Layer (LSL) (Kothe, 2014) was used to synchronize behavioral and neural responses and record them into a single file. Researchers also developed and publicly shared a low-latency pipeline in Java programming language to parse in the real-time measured EEG data from the Emotiv EPOC+ application development interface (API) and parse out in LSL (O’neil, 2019). App-LabRecorder (2019) software was utilized as a unified recorder to collect and save behavioral and neural time series in a single file with the.xdf file extension. XDF is an open-source and general-purpose file format designed in tandem with LSL and supports LSL protocol features. The file extension is compatible with both MATLAB and Python languages and provides a convenient data structure container to record various data modality streams, concurrently. Researchers have publicly shared the experimental setup under open-source terms and conditions for other researchers [Borhani (2019) for free sharing]. The developed platform with all its software dependencies were installed on a computer in the clinics at United Kingdom-ADC and on a workstation in Aging, Brain, and Cognition Laboratory, United Kingdom College of Medicine.

### Data Analysis

#### Resting-State EEG Preprocessing and Frequency Analysis

The impedance between each electrode and scalp were kept below 10 KW. EEGLAB software library (Delorme and Makeig, 2004) was employed for preprocessing and artifact removal. EEG signals were re-referenced to the average and band-pass filtered between 0.5 and 46 Hz. EEG recordings were analyzed for eye blinks and muscle artifacts. The artifact subspace reconstruction (ASR) algorithm (Kothe and Makeig, 2013; Gabard-Durnam et al., 2018) was implemented in the EEGLAB software and employed to cope with channel noise and artifacts. ASR algorithm is an advanced method that allows the detection and reconstruction of noisy and artifactual chunks of EEG signals. Makoto’s EEG preprocessing pipeline (Makoto, 2018), along with Pernet et al. (2020) recommendations for EEG processing, were followed to process the EEG signals. Channels that were flat for more than 5 s, or with abnormal high/low peaks were deleted and removed. ASR finds the cleanest part of data as the calibration data by applying a 0.5-s sliding window principal component analysis (PCA) on the continuous EEG data to classify principal components into high variance (20 standard deviations the calibration data) or normal variance. By detecting high variance chunks of signals, ASR reconstructs the high variance subspace using the normal calibration chunks. A minimum of 60 s from each participant’s data after channel noise and artifact removal was kept for analysis.

A Fast Fourier Transform (FFT) on 4-s epochs of EEG yields a 0.25 Hz frequency resolution over the frequency span of 1–46 Hz. A sliding window of 4-s window length with a 2-s overlap was used to minimize the effect of the windowing of FFT procedure. The band power in delta (d:1–4 Hz), theta (q: 4–8 Hz), alpha (a: 8–13 Hz), beta (b: 13–28 Hz), and gamma (g: 28–46 Hz) frequency bands from all 14 EEG electrodes were computed. The absolute band power in each frequency band as well as magnitude-squared inter-channel coherence is computed for the eyes-closed resting-state session.

#### EEG Coherence Analysis

An electrode-by-electrode analysis was conducted to correlate rEEG band powers and magnitude-squared coherence with the memory task’s performance measures. The approach helped to identify correlations between band-delimited power in all electrodes and the memory task performance. Pearson correlations between EEG band power and task performance presented the relationship, and significant correlations depicted the participants’ dominant pattern. The recorded EEG data were analyzed using custom scripts in the MATLAB language (MATLAB, 2019). For each electrode, the correlation between the participants’ behavioral measures of the memory task (mean reaction time and mean accuracy score) and mean power bands in the delta (d:1–4 Hz), theta (q: 4–8 Hz), alpha (a: 8–13 Hz), beta (b: 13–28 Hz), and gamma (g: 28–46 Hz) frequency bands were calculated using Pearson correlation. Additionally, the correlations between the inter-electrode coherence network during resting-state and the and memory task performance measures were analyzed. MATLAB connected topoplot toolbox (Namburi, 2020) was used to illustrate pairs of inter-channel coherence with a significant correlation between resting-state and task performance measures. To explore connectivity between all pairs of EEG electrodes in all oscillation bands, the magnitude-squared coherence was used as the connectivity estimation metric obtained by:

$$C\,o\,h_{x\,y}\,\left(f\right)=\frac{{\left|G_{x\,y}\,\left(f\right)\right|}^{2}}{G_{x\,x}\,\left(f\right).G_{y\,y}\,\left(f\right)}\;\left(1\right)$$

Where *G*_*xx*_ and *G*_*yy*_ are autospectral density of channels x and *y* and *G*_*xy*_ is the cross-spectral density between EEG signals on channel x and channel *y*. Autospectral and cross-spectral density are functions of frequency. The magnitude coherence between two channels is an estimate showing the predictability of information from one channel using the other channel’s data. We identified the neural correlates of resting-state EEG that correspond to task-dependent behavioral scores (see Figure 1) in a population of aging adults with normal cognition.

## Results

Using a wireless EEG setup in both laboratory and clinics, an 11-min visual working memory task (modified delayed matched to sample) was used to investigate rEEG surrogates of memory performance. Every participant’s reaction-time (seconds) and accuracy score (correct percentage) during the working memory task were analyzed. Furthermore, correlations between the behavioral measures and neural measures were conducted.

### Behavioral Results

#### Accuracy of Working Memory Retrieval

**FIGURE 2:**
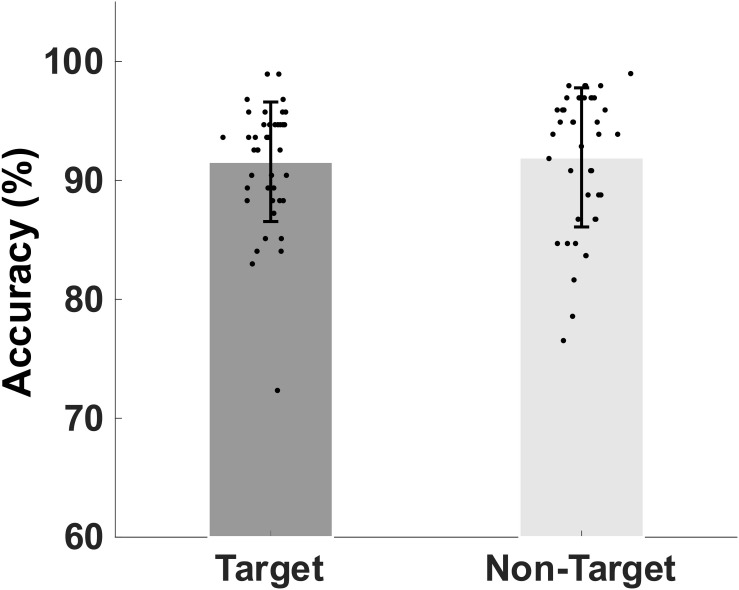
Bar plot showing group mean percent and standard deviation of the mean accuracy of retrieval of target match and non-target, non-match visual stimuli.

The mean and standard deviation (SD) of the older participants’ percentage accuracy in correctly identifying the memory target and non-target stimuli were shown in Figure 2. The mean (SD) accuracy of the correct responses to target and non-target stimuli are 91.56 (5.02)% and 91.93 (5.84)%, respectively.

#### Reaction Times of Working Memory Retrieval

**FIGURE 3:**
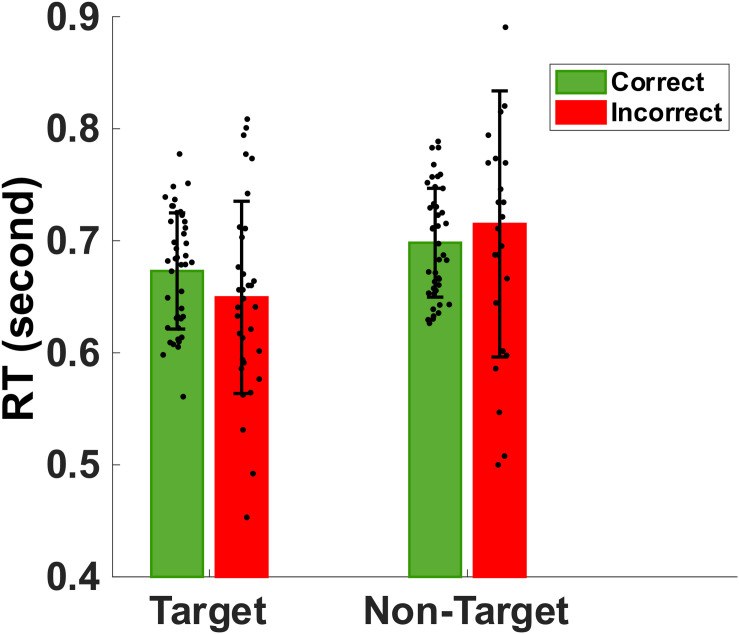
Bar plot showing the group mean and standard deviation of the reaction times to target match and non-target distractors during correct (green) and incorrect (red) trials. The normal older adults were significantly faster in identifying memory targets than non-targets (*p* < 0.05).

Figure 3 shows the mean and standard deviation of the reaction times to memory target match and non-match. Mean (SD) of correct reaction times to target and non-target images were 673 (51) ms and 698 (48) ms, respectively. The non-parametric Kruskal–Wallis significance test revealed a significantly faster correct reaction to target-match compared to non-match (*p* < 0.05). Also, we extracted the mean incorrect reaction time to target and non-target (false alarm) stimuli. Mean (SD) of mean incorrect reaction time to target and non-target images are 649 (85) ms and 715 (118) ms, respectively. Non-parametric Kruskal-Wallis significance test revealed a significantly slower reaction to non-target non-matched (false alarm) images compared to target images (p < 0.05).

#### Sex Differences in Accuracy and Reaction Times

**FIGURE 4:**
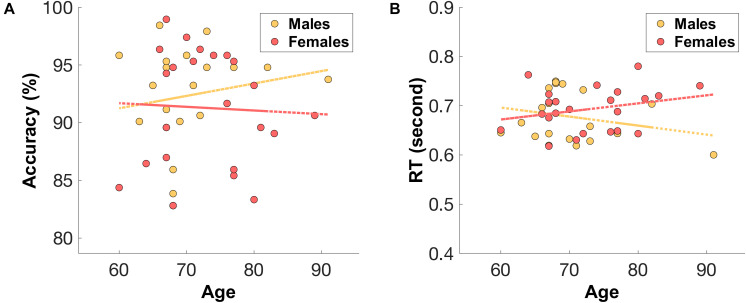
The distribution of **(A)** mean accuracy score and **(B)** mean reaction time (RT) across age for both male (orange) and female (red) groups.

The male group (*n* = 20, median = 68, IQR = 5.5) were relatively younger than the female group (*n* = 23, median = 72, IQR = 10). Unsurprisingly, males showed a relatively higher accuracy (mean = 92.34%, SD = 4.32%) and faster reaction times (mean = 677 ms, SD = 51) (see Figure 4) compared to those of older females (accuracy: mean = 91.28%, SD = 4.97%, RT: mean = 693 ms, SD = 43).

### Resting-State Alpha Band During Eyes-Closed and Eyes-Open

**FIGURE 5:**
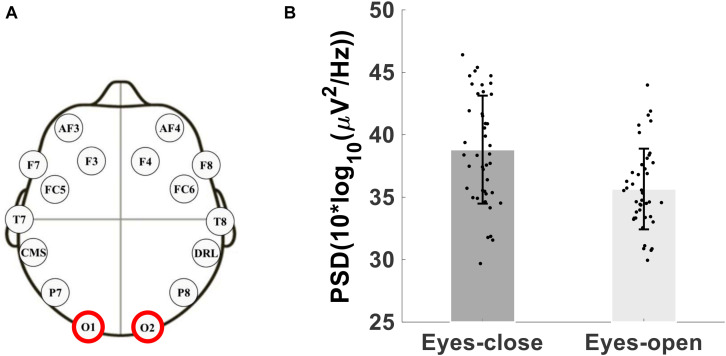
**(A)** Montage of the 14-channels wireless EEG headset, **(B)** Bar plot showing the group average and standard deviation of the alpha band power over occipital sites (O1 and O2 electrodes) during eyes-open and eyes-closed.

**FIGURE 6:**
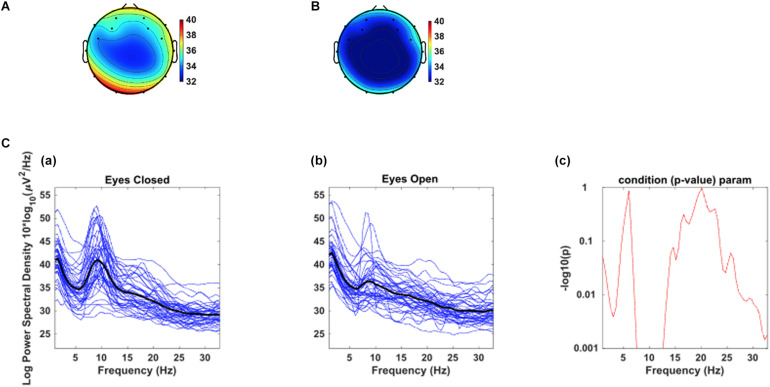
The corresponding dorsal view topographic distribution of the alpha wave (8–13 Hz), during resting-state **(A)** eyes-closed and **(B)** eyes-open (Top panel). C. Average power spectral density at combined occipital sites (O1 & O2) during eyes-closed **(A)** and eyes-open **(B)**; The black color curves show the average occipital power spectral density of all participants, and the blue curves show individuals’ power spectral density. **(C)** the point by point confidence level shown by p-value in the older adults.

As a test of quality reassurance of our EEG data, we analyzed the eyes-closed and eyes-opened alpha bands because increased alpha activity under eyes closed is well established in the literature (as described in the introduction). We examined the group average and the group standard deviation of the alpha band power over the occipital sites (O1, O2) during resting-state (see Figure 5). The group mean ± SD during the resting-state eyes-closed is 38.80 ± 4.32 (μ*V*^2^/*Hz*) and during resting-state eyes-open is 35.65 ± 3.23 (μ*V*^2^/*Hz*). Using non-parametric Kruskal-Wallis significance test, the average occipital alpha during the resting-state eyes close is significantly larger than the eyes-open (*p* = 3.6×10^−4^). Figure 6 illustrates average power spectral density during resting-state eyes opened and eyes closed and the corresponding topographic distribution of the alpha frequency spectrum (8–13 Hz), showing the increased occipital alpha during eyes-closed compared to eyes-open resting-state EEG.

#### Eyes-Closed Resting-State EEG and Task-Related Behavior

##### rEEG and Reaction Times

**FIGURE 7:**
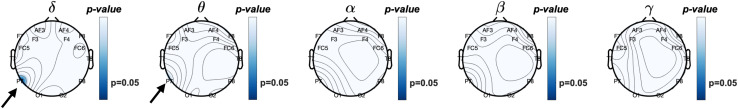
Topographical plot of Pearson correlations between resting EEG (Eyes-closed) relative frequency band power and mean reaction times during working memory task. Significant negative correlation was observed at the P7 (left parieto-occipital site) at delta (δ) frequency band (*p* < 0.05) and approaching significant (*p* = 0.068) theta (θ) band power. In other words, increased δ and θ frequencies power observed in the left posterior brain region is correlated with faster reaction times. Reaction times did not correlate with higher frequency band power, i.e., α, β, nor γ.

Since eyes-closed rEEG is gaining momentum as easy biomarker for cognitive decline, we examined Pearson correlations between rEEG relative frequency band power recorded during the resting-state eyes-closed session before the memory task and mean behavioral results during the working memory task. Figure 7 show the topographical distribution of the Pearson correlations between eyes closed rEEG relative frequency band power and reaction times during the memory task. The significant correlations between rEEG power spectral density in each frequency band on 14 EEG sites and the individuals’ performance in the working memory task were investigated. A significant negative correlation was found between individuals’ mean reaction times during the memory task and the delta band power (ρ(43) = −0.31,p < 0.05). The correlation is mostly focused over the left parietal site over the P7 EEG electrode.

With approaching significance, higher activities in the theta band over the left parietal correlates with faster reaction time (ρ(43) = −0.28,p = 0.07). As shown in Table 1, brainwave EEG during resting did not correlate with mean reaction times in the higher frequency bands in alpha, beta, and gamma bands.

**TABLE 1:** Correlations between eyes-closed resting-state EEG and mean reaction time.

**Frequency band (Hz)**	**Electrode (location)**	**Pearson correlation (**ρ)	***p***
delta (1–4 Hz)	P7 (left parieto-occipital)	−0.31	0.042*
theta (4–8 Hz)	P7 (left parieto-occipital)	−0.28	0.068

*indicates significant p value at the 0.05 level.

##### rEEG and Accuracy

**FIGURE 8:**
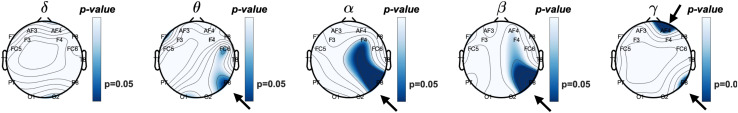
Topographical plot of Pearson correlations between relative frequency band power of resting eyes-closed EEG and Accuracy (% correct) of the working memory task.

Next, we explored a potential rEEG neuromarker of individuals’ mean accuracy scores (percentage of correct responses) during a short-term memory task. Figure 8 reveals the topographical distribution of Pearson correlations between the eyes-closed rEEG and mean percent accuracy during the memory task. A significant negative correlation (ρ(43) = −0.42,p < 0.01) was found between individuals’ EEG frequency band power in the beta band over the right parietal site (P8 electrode).

Table 2 shows Pearson correlations and the significance level in terms of p-value for the EEG frequency bands and EEG electrodes. The significance level of correlations between eyes-closed resting-state and mean accuracy scores were lower than 0.05. The overall distribution of the Pearson correlations in all frequency bands and all EEG electrode sites suggests that lower brain activity over the right parietal site in alpha and beta bands as well as the right frontal site in the gamma band during eyes-closed resting-state was significantly correlated with higher ability to correctly distinguish between target and distractor images in a short-term memory task. Lower alpha and beta activities of rEEG over right parietal areas during eyes-closed were found to be indicators of higher accuracy score.

**TABLE 2:** Correlations between eyes-closed resting-state EEG and mean accuracy score.

**Frequency band (Hz)**	**Electrode (location)**	**Pearson correlation (**ρ)	***p***
delta (1–4 Hz)	O2 (right occipital)	−0.29	0.058
theta (4–8 Hz)	F7 (left frontal)	−0.31	0.043*
	O1 (right occipital)	−0.28	0.064
	P8 (right parietal)	−0.31	0.041*
	FC6 (right frontocentral)	−0.29	0.059
alpha (8–13 Hz)	P8 (right parietal)	−0.39	0.0095**
	F4 (right frontal)	−0.28	0.068
beta (13–28 Hz)	P8 (right parietal)	−0.42	0.0054**
gamma (28–46 Hz)	P8 (right parietal)	−0.32	0.033*
	AF4 (right frontal)	−0.33	0.032*

*indicates significant p value at the 0.05 level; **indicates significant *p* < 0.01.

##### Magnitude-Squared Coherence of the Fronto-Parietal Sites Subserving Reaction Times

**FIGURE 9:**
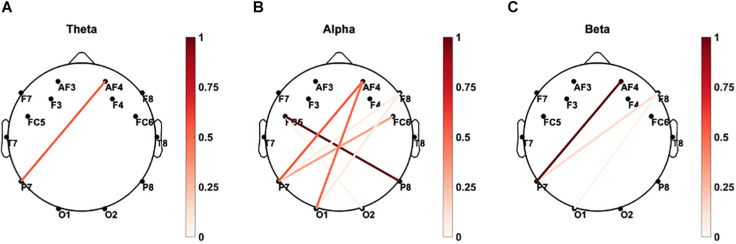
Brain connectivity graphs showing the significant correlations between paired EEG channels’ magnitude-squared coherence during the eye-closed resting-state and mean reaction time in **(A)** delta, **(B)** theta, **(C)** alpha, **(D)** beta, and **(E)** gamma bands. The color designates the level of correlation between paired coherence and the reaction times were significant (p < 0.05). Increased alpha coherence between the left frontal and right posterior sites correlates with increased reaction time.

The coupling between brain sites within the delta, theta, alpha, beta, and gamma frequency bands based on the magnitude-squared coherence algorithm was explored. As demonstrated in Figure 9, significant correlations (p < 0.05) between paired eyes-closed rEEG magnitude-squared coherence in frequency band oscillations during and mean reaction time were extracted. Significant differences were mainly found in the alpha band in the EEG inter-channel coherence network. Increased alpha coherence between the right parietal and the left frontal sites correlates with increased reaction time.

##### Coherence of the rEEG at Parietal Sites in the Delta and Theta Bands Correlates With the Mean Percent Accuracy

**FIGURE 10:**
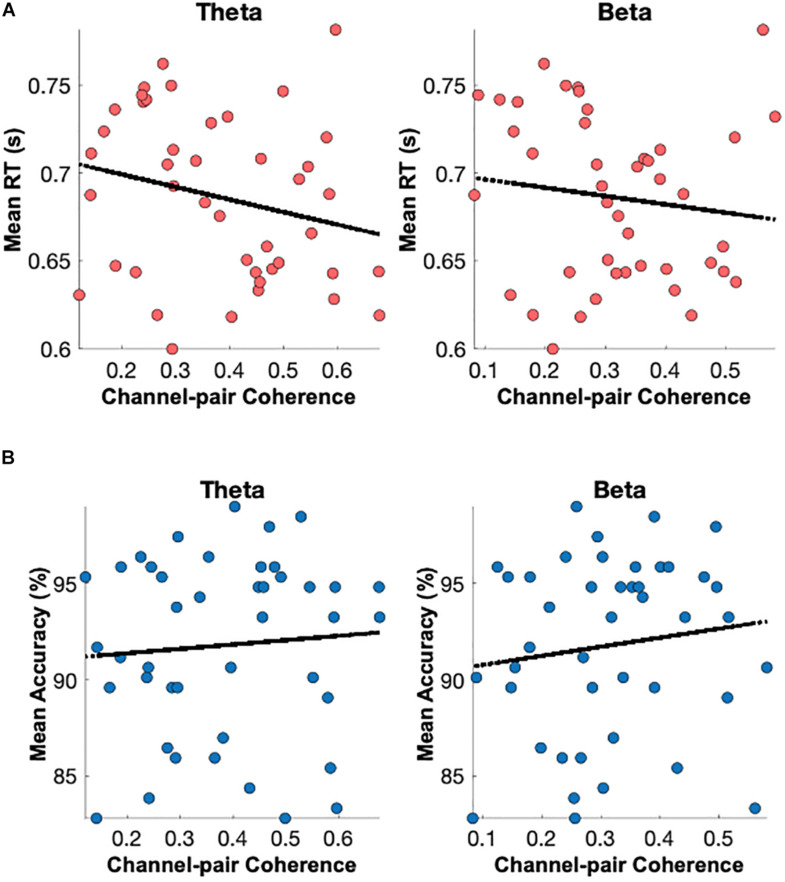
Brain connectivity graphs showing the significant correlations between mean percent accuracy and paired rEEG channels’ coherence during the eye-closed in **(A)** delta, **(B)** theta, **(C)** alpha, **(D)** beta, and **(E)** gamma bands. The color designates the level of correlation between all paired coherence and the corresponding measure where the observed correlation was significant (p < 0.05). Significant increased of frontal delta and theta coherence correlates with better memory accuracy.

As demonstrated in Figure 10, significant correlations (p < 0.05) between paired rEEG magnitude-squared coherence in oscillations during frequency band and mean accuracy score were extracted. Significant correlations were found in the theta and delta bands for the rEEG inter-channel coherence network. Increased frontal contralateral coherence between frontal and temporal sites is positively correlated with increased mean accuracy scores during memory retrieval. No significant correlations were noted in the inter-channel coherence network in the beta band and mean accuracy score.

## Discussion

### Summary of the Findings

We report several new findings to explore the clinical-friendly eyes-closed rEEG and healthy older individuals’ working memory capacity. First, working memory retrievals’ accuracy is correlated with higher frequency brain oscillations (beta and alpha) at local and distant neural circuits in the right hemisphere support. Second, faster memory retrieval is significantly correlated with increased delta and theta band powers over the left parietal sites. Also, increased coherence between the right parietal site and the left frontal area is associated with slowed memory retrieval speed.

### Accuracy of Working Memory Retrievals and Gamma, Beta, and Alpha

Before we test our hypothesis, we analyzed memory performance data and alpha eyes closed and open data to make sure the quality of our performance and EEG data. We found that accuracy is approximately 91% to correctly identify one of the two memory targets and non-target distractors in the current working memory task in older adults. In a slightly easier version of the task (only one memory target), the accuracy was 92% and 97% (Lawson et al., 2007). Eyes-closed (EC) evoked larger alpha powers than eyes open (EO) has been well documented in the literature. Interestingly, the difference between EC and EO are reduced in patients with MCI and AD (Wan et al., 2019). The results from the current research are consistent with previous literature of rEEG in healthy older adults.

We report the significant correlations between memory retrieval accuracy and rEEG band power. Specifically, increased alpha and beta bands over the right parietal and gamma in the right frontal sites, were significantly correlated with poorer accuracy during the memory task. The right parietal site within alpha and beta bands showed even a higher relationship with the mean accuracy compared to the gamma band.

### Reaction Times and Delta/Theta Band Powers

Interestingly, memory retrieval of non-match distractor objects took longer than target objects (see Figure 3). Pearson correlation was analyzed between neural oscillations in different frequency bands recorded with a wearable EEG headset during eyes-closed resting-state and the participants’ mean reaction time and mean accuracy score during a delayed visual target matching task. The results suggest that rEEG in specific brain sites and specific rhythms are significantly correlated with working memory performance measures. Researchers discovered that the analysis of EEG signals during resting and conscious state of mind carries a predictive utility to tell apart behavioral performance associated with short-term memory performance. Results of significant correlations of rEEG band powers and individuals’ mean reaction time indicate that a faster reaction in target matching memory task to significantly correlates with higher delta and theta rhythms over left parietal sites (Figure 7). Additionally, we discovered that rEEG over right parietal and right frontal sites carried valuable information about the mean accuracy of target recognition in the memory task. Lower brain activities in alpha, beta, and gamma bands over the right parietal region correlate well with a higher accuracy score. These results suggest that rEEG over the parietal can be a potential biomarker, reflecting memory retrieval accuracy among normal aging adults.

The current findings are consistent with the literature that higher theta and lower beta, which results in higher theta/beta ratio before, is an indicator of memory capacity. Heister et al. (2013) studied neural associates of working memory during resting-state captured by MEG signals, which found that right frontal and parietal cortex delta/theta power were inversely correlated with three-back working memory performance. Those results demonstrated that individuals with poor accuracy in working memory showed larger increases in right posterior frontal and parietal delta/theta in resting-state condition. An individual’s working memory also requires input from attention. A new study highlighted the predictive utility of resting-state EEG and investigated neural correlates of vigilance score and response time during varying-duration sessions of sustained attention to response task (SART) (Torkamani-Azar et al., 2020). The results indicated an increase in the left central and temporal gamma, and upper beta during rest predicts slower reaction time.

### Selective Neural Communications Within the Working Memory Network *via* Frequency Oscillations and Coherence

The nature of neural coding in mammalian brains, which may support selective communication, are not fully understood. Research has postulated that the structure of oscillations varies in time and space according to behavioral state. Multiplexing implemented through periodic modulation of firing-rate population codes enables flexible reconfiguration of effective connectivity. Memory retrieval is a dynamic process continuously regulated by both synaptic and intrinsic neural mechanisms, e.g., intrinsic excitability, synaptic plasticity, and interactions among brain areas (Chen et al., 2020). The findings suggest that neural dynamics underlying accuracy are different from those undeserving memory retrieval speed.

By analyzing spectral coherence between paired EEG sites, we reported increased connectivity between resting EEG sites and working memory accuracy and reaction times. Literature has suggested the significance of coherence between frontal and posterior brain areas as an indicator of brain overall cognitive function. In a comprehensive review of the literature (Babiloni et al., 2016), changes in the connectivity network between different brain areas, including the frontal and parietal areas, have been observed among people with MCI and AD. The research has demonstrated that the coherence between frontoparietal regions in the theta band would correlate with the working memory performance-related measures on the Digit Span (Tóth et al., 2012). Fleck et al. (2016) studied resting-state EEG in search of a neuromarker of cognitive decline, and they observed a positive correlation between the delta and the beta coherence within the frontal and posterior regions and performance on measures of memory and executive function in older adults.

### Implications and Limitations

Findings suggest an increased resting eyes-closed delta and theta bands at the left parietal site are associated with faster speed, while increased alpha and delta at right parietal site is associated with reduced accuracy during working memory. Increased coherence between the right parietal and the left frontal sites correlates with slowed reaction time. The frontal and posterior dynamics of resting EEG is associated with the “*accuracy and speed trade-off*” during working memory in healthy older adults.

Importantly, the wireless headset is a beneficial, pre-screening tool utilized at physician offices before sophisticated biomarkers tests in various clinical populations. Deficits in working memory are common in amnestic mild cognitive impairment (MCI), an early stage of AD, and related dementia (ADRD). Currently, the AD biomarkers for early diagnosis of mild cognitive impairment are not only expensive to measure, but also involve invasive and time-consuming neuroimaging, laboratory examinations, and cognitive assessments. Depending on the degree of change, the quantitative analysis of cognitive processing changes may provide evidence of pathologic cognitive changes beyond age-related decline, MCI, and ADRD.

The study is a preliminary stage to test the utility of the resting-state EEG using a wearable headset as a pre-screening methodology for cognitive measures besides other well-established behavioral and cognitive questionnaires. There are both advantages and disadvantages to current rEEG approaches, which gauge working memory capacity. Currently, clinicians rely on well-established but time-consuming neuropsychological testing as a proxy to identify changes in brain networks in the aging population. Different cognitive tasks such as working memory and selective attention have been used to enumerate the rate of cognitive decline measured by accuracy and reaction time, which has been shown to provide a reliable cognitive decline measure. Cognitive testing is less sensitive for the small changes in memory that accumulate overtime but can identify the changes’ summation when the effect is significant. However, cognitive deficiency may not be appeared and diagnosed at the onset of AD, motivating the discovery of non-invasive and inexpensive-to-collect neuromarker, i.e., a neuromarker showing altered brain connectivity across interconnected networks of the brain.

A noted limitation in the present study was a small sample size (*n* = 43). As such, further research is necessary to understand the underlying neural mechanisms. Additionally, a follow-up study should include a longitudinal approach that includes a large number of healthy, aging adults. With the wearable and wireless EEG headset that is non-invasive and easy-to-use, the current study offers an affordable option for large-scale repeated recording in the clinics. Furthermore, research efforts should focus on evaluating EEG neuromarkers in patients with amnestic MCI, or those in dementia. Future investigation is needed to evaluate the associations and correlations of rEEG with other neural degenerative biomarkers such as A-beta, p-tau, or cortical atrophy in MRI scans. Overall, brief resting and task protocol using wearable EEG headset has demonstrated great potential for gauging working memory capacity, a sensitive and fast screening tool for cognitive impairment risk. Our current findings indicate the need for future large-scale validation to understand individual measurements of rEEG frequency and coherence features.

## Conclusion

We report that the frontal and posterior dynamics of resting EEG is associated with the “*accuracy and speed trade-off*” during working memory in healthy older adults. The Bluegrass protocol recorded resting EEG oscillations under 3 min and likely to be used as fast surrogate markers for assessing individual working memory capacity.

## Data Availability Statement

The raw data supporting the conclusions of this article will be made available by the authors, without undue reservation.

## Ethics Statement

The studies involving human participants were reviewed and approved by the Institutional Review Board (IRB) of the University of Kentucky, Lexington, KY, United States. The patients/participants provided their written informed consent to participate in this study. Written informed consent was obtained from the individual(s) for the publication of any potentially identifiable images or data included in this article.

## Author Contributions

YJ, GJ, SB, and XZ designed the research. MK, KG, and GJ conducted the neuropsychological and EEG data collection and behavioral data analysis. SB, YJ, FY, and XZ performed the EEG and statistical analysis. SB, YJ, and XZ wrote the manuscript. All authors contributed to the article and approved the submitted version.

## Conflict of Interest

The authors declare that the research was conducted in the absence of any commercial or financial relationships that could be construed as a potential conflict of interest.
